# Awe Walks Improve Quality of Life, Attention, Orientation, Mood & Social Behaviour in Dementia: A One Year Follow‐Up

**DOI:** 10.1002/alz.083585

**Published:** 2025-01-09

**Authors:** Akshata Sheth, Cathlyn Bennett

**Affiliations:** ^1^ Christ University, Bangalore India

## Abstract

**Background:**

Research has consistently shown decreased quality of life (QoL) in people with dementia, with predictors of QoL ranging from education to emotional status. This study, along with a one year follow‐up study, investigated the impact of Awe Walks as an intervention targeting emotional status for the first time in dementia. Awe—a positive emotion elicited when in the presence of vast things not immediately understood—promotes social connection and fosters well‐being by encouraging a “small self”.

**Method:**

Participants with dementia between the ages of 60 and 85 took biweekly 15‐min outdoor walks for 4 weeks; a total of 53 participants were matched and randomly assigned either to an awe walk group, which oriented them to experience awe during their walks, or to a waitlist control group. Pre‐ and post‐intervention measures of QoL, cognitive functioning, behavioural pathology relevant to daily functioning, and clinical global impression were completed.

**Result:**

Compared to degenerative deterioration in controls, individuals who participated in the Awe Walk intervention exhibited greater improvements in QoL, attention, orientation, mood, and social behaviour.

**Conclusion:**

These results suggest cultivating awe enhances positive emotions that improve quality of life and diminishes negative emotions that hasten decline. An intervention such as this, cost‐effective and simply executed in the long term, has important implications for providing quality care to this population served by neuropsychologists.

